# Introducing the Ensemble-Based Dual Entropy and Multiobjective Optimization for Hydrometric Network Design Problems: EnDEMO

**DOI:** 10.3390/e21100947

**Published:** 2019-09-27

**Authors:** Jongho Keum, Frezer Seid Awol, Jacob Ursulak, Paulin Coulibaly

**Affiliations:** 1 Department of Civil Engineering and School of Geography and Earth Sciences, McMaster University, Hamilton, ON L8S 4L8, Canada; 2 Department of Civil Engineering, McMaster University, Hamilton, ON L8S 4L8, Canada; awolf@mcmaster.ca; 3 Department of Civil Engineering, McMaster University, Hamilton, ON L8S 4L8, Canada; ursulajp@mcmaster.ca; 4 Department of Civil Engineering and School of Geography and Earth Sciences, McMaster University, Hamilton, ON L8S 4L8, Canada; couliba@mcmaster.ca; 5 United Nations University Institute for Water, Environment, and Health, Hamilton, ON L8S 4L8, Canada

**Keywords:** EnDEMO, hydrometric network, network design, monitoring, entropy, ensemble, uncertainty, information theory, multiobjective optimization

## Abstract

Entropy applications in hydrometric network design problems have been extensively studied in the most recent decade. Although many studies have successfully found the optimal networks, there have been assumptions which could not be logically integrated into their methodology. One of the major assumptions is the uncertainty that can arise from data processing, such as time series simulation for the potential stations, and the necessary data quantization in entropy calculations. This paper introduces a methodology called ensemble-based dual entropy and multiobjective optimization (EnDEMO), which considers uncertainty from the ensemble generation of the input data. The suggested methodology was applied to design hydrometric networks in the Nelson-Churchill River Basin in central Canada. First, the current network was evaluated by transinformation analysis. Then, the optimal networks were explored using the traditional deterministic network design method and the newly proposed ensemble-based method. Result comparison showed that the most frequently selected stations by EnDEMO were fewer and appeared more reliable for practical use. The maps of station selection frequency from both DEMO and EnDEMO allowed us to identify preferential locations for additional stations; however, EnDEMO provided a more robust outcome than the traditional approach.

## 1. Introduction

The successful planning and completion of a typical water resources management project relies primarily on the collection of dependable hydrological information from an adequate hydrometric network [1,2,3,4]. While advances in remote sensing technologies provide an alternative to traditional ground-based monitoring networks, nonetheless reliable in-situ observations are still needed to fill in gaps in remotely sensed datasets and verify the accuracy of remote sensing measurements [5]. However, aging infrastructure and financial constraints have contributed to a decline in the spatial density of existing ground based hydrometric networks in many countries [6,7,8]. This in turn necessitates the need to determine an optimal hydrometric network by defining a sufficient number of monitoring stations and their optimal spatial distribution.

The application of the entropy concept in information theory for the field of hydrometric network design problems is well reviewed by Keum et al. [4]. Many of the data-driven hydrometric design methods, such as entropy applications, require hydrological time series for both existing and potential (non-existing but candidate) station locations [5,9,10,11,12,13,14,15,16,17]. As a result, synthetic time series must be generated for each potential station. In most of the previous studies, regionalization methods, such as spatial interpolation like inverse distance weighting (IDW) combined with drainage area ratio (DAR) and rainfall runoff models, were applied to generate time series.

For most of the entropy-based hydrometric network design problems (e.g., [5,13,14,15,18,19]), synthetic (or simulated) time series from such methods have been deterministic; therefore, any outcomes which are driven from those deterministic time series can be considered uncertain, as the uncertainty of the synthetic time series was not accounted for. A previous study [20] suggested the ensemble entropy. However, the concept was limited to changing various bins of a histogram when calculating entropy measures; input data uncertainty was not accounted for in that approach. Hence, this study aims to develop a methodology which combines the generation of ensemble time series and utilizes them as an input of the sophisticated, entropy-based, multiobjective optimization tool. Specific objectives throughout this study are as follows:

(1) Evaluate the existing hydrometric networks by applying the entropy-based transinformation analysis;
(2) Identify potential station locations in the study area and define the simulated time series for each station;
(3) Apply the traditional, deterministic, entropy-based hydrometric network design approach to find the optimal networks;
(4) Apply the proposed ensemble-based design approach;
(5) Compare the optimal networks and suggest the locations where the additional monitoring is recommended.

## 2. Study Area and Data Preparation

**Figure 1:**
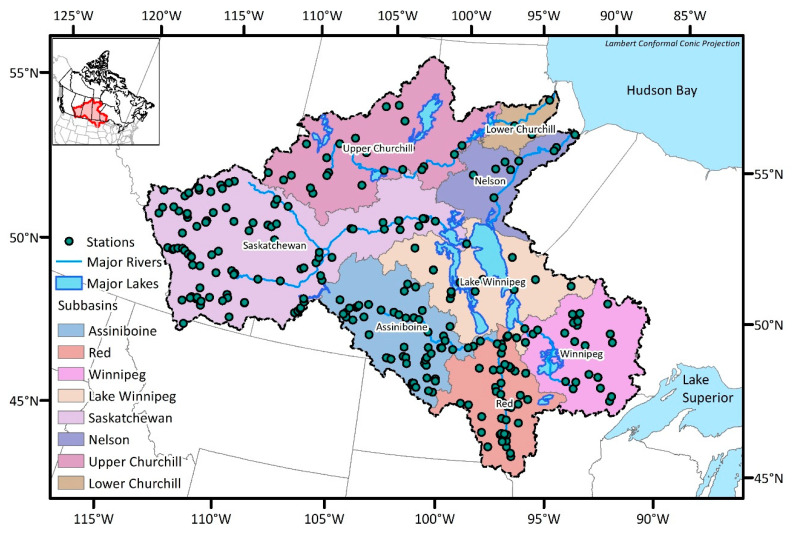
Nelson and Churchill river basins and the existing streamflow gauging stations.

The study area covers both the Nelson River Basin and the Churchill River Basin, which are adjacent basins to each other and are herein referred to as the NCRB (see Figure 1). The drainage area of the NCRB is 1.4 million square kilometers, which makes it the third largest basin in North America. The NCRB is bounded by the Rocky Mountains to the west and the Lake Superior to the east. It drains, generally, from south to north and from west to east, ultimately discharging to the Hudson Bay at the NCRB’s northeastern end. Elevation within the NCRB ranges from the sea level at the Hudson Bay outlet to 3550 m above mean sea level in the Rocky Mountains. The major river systems are the Nelson and Churchill Rivers; the former can be broadly sub-divided into six major sub-basins: Assiniboine, Red, Winnipeg, Lake Winnipeg, Saskatchewan, and Nelson, while the later consists of two sub-basins: Upper Churchill and Lower Churchill. The topography of the NCRB is dominated by forest and wetland, which constitutes nearly 70% of the land cover [21]. The central prairie region is the driest zone of the NCRB, with average annual precipitation levels of less than 320 mm. The wettest zones of the watershed are found at the eastern and western extents, near the Rocky Mountains and Lake Superior respectively, which receive an average annual precipitation of more than 750 mm. Annual average temperatures vary from 6 °C in the south to −6 °C north. In the winter, average temperatures vary from −4 °C to −22 °C. 

**Figure 2:**
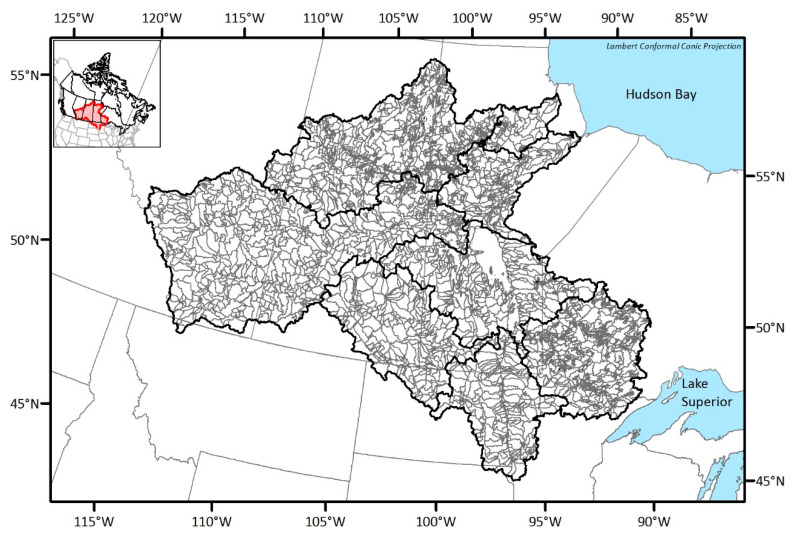
Delineated catchments for locating potential stations.

There are 267 gauges which monitor streamflow in the NCRB, and those monitoring stations are relatively well distributed throughout the basin. Given that the drainage area of the NCRB is roughly 1.4 million square kilometers, the average coverage of each station becomes 5200 km^2^. The minimum network guideline by World Meteorological Organization (WMO) [22] recommends 1875 km^2^ per station for streamflow monitoring in interior plains. This shows that the NCRB currently requires the installing of new monitoring stations to collect more representative information from this large basin. The number of existing stations and the number of catchments associated with each sub-basin are summarized in Table 1. For the streamflow monitoring, catchment topology is one of the important aspects: hence, the outlets of the catchments were selected as potential station locations in this study (see Figure 2). The number of catchments which is equivalent to the number of potential stations is 2693. This study follows the delineation result which was accomplished during the Hydrological Prediction for the Environment (HYPE) modeling process [23], which will be described in Section 4.

## 3. Background

### 3.1. Information Theory

The entropy concept in information theory has been used to measure the amount of information from a given system [24]. Entropy itself refers to uncertainty. If additional information is given to a system, then some of the uncertainty associated with the additional information becomes certain. In this way, the information theory relates uncertainty to entropy. There are several basic terms of entropy used in this study: marginal entropy, joint entropy, and total correlation.

Marginal entropy is the amount of information in a variable. In network design problems, this is equivalent to the amount of information that a single station can provide.

(1) $$H\left(X\right)=-\overset{n}{\underset{i=1}{\sum }}p\left(x_{i}\right)\log_{2}p\left(x_{i}\right)$$

where *H*(*X*) is the marginal entropy of a station *X* (bits); $p\left(x_{i}\right)$ is the occurrence probability of $x_{i}$ at station *X* for the *i*-th bin; and *n* is the total number of bins in a histogram distribution. The amount of information from multiple variables, i.e., information from several stations or a hydrometric network, can be measured in a similar way. The joint entropy employs a joint probability.

(2) $$H\left(X_{1},\;X_{2},\cdots ,\;X_{N}\right)=-\overset{n_{1}}{\underset{i_{1}=1}{\sum }}\overset{n_{2}}{\underset{i_{2}=1}{\sum }}\cdots \overset{n_{N}}{\underset{i_{N}=1}{\sum }}p\left(x_{1,i_{1}},x_{2,i_{2}},\cdots ,x_{N,i_{N}}\right)\log_{2}p\left(x_{1,i_{1}},x_{2,i_{2}},\cdots ,x_{N,i_{N}}\right)$$

where $H\left(X_{1},\;X_{2},\ldots ,\;X_{N}\right)$ is the joint entropy of *N* stations, $n_{1},\;n_{2},\cdots ,n_{N}$ are the number of bins of a network with *N* stations [25]. When the variables (stations) are independent, the joint entropy is equal to the sum of the marginal entropy of stations. To the exclusion of completely independent variables, total correlation, which is given by the difference between the sum of the marginal entropy and the joint entropy, has a positive value.

(3) $$C\left(X_{1},X_{2},\cdots ,X_{N}\right)=\;\overset{n}{\underset{i=1}{\sum }}H\left(X_{i}\right)-\;H\left(X_{1},X_{2},\cdots ,X_{N}\right)$$

where $C\left(X_{1},X_{2},\cdots ,X_{N}\right)$ is the total correlation of *N* stations. While the total correlation represents the duplicated information of multiple stations, transinformation (TI) yields that of two stations.

(4) T(A,B)=H(A)−H(A|B)=H(B)−H(B|A)=T(B,A)

where *T*(*A*,*B*) is the TI value between variables *A* and *B*. *H*(*A*) is the marginal entropy of *A*, which is a measure of the information content provided to a system by variable *A*. *H*(A|B) is the conditional entropy of *A* given *B*, which refers to the amount of information content in *A* that is provided by already knowing *B*.

### 3.2. Transinformation Analysis

TI analysis employs information theory to evaluate the target hydrometric network by ranking monitoring stations based on their relative importance within the network [12,26,27]. The TI value of each station is calculated such that the variable A is a time series at a single station of interest, while the variable B represents a synthetic time series generated from multiple linear regression of the remaining stations within the network [26]. Once TI values are determined for each station, they are normalized using the following equation.

(5) $$X_{i}=\frac{x_{i}-x_{min}}{x_{max}-x_{min}}$$

where $X_{i}$ is the value being normalized and $x_{min}$ and $x_{max}$ are the minimum and maximum values within the corresponding dataset. Once the normalized TI index of each existing station is determined, the index values are spatially interpolated to construct the TI index maps.

A station with a lower TI index represents a more unique information content and is of greater value to the network. Conversely, stations with a higher TI index may share large amounts of information with neighboring stations. They could potentially be considered redundant and less important to the network.

### 3.3. Dual Entropy and Multiobjective Optimization (DEMO)

The efficient design of an optimal hydrometric network will minimize information redundancies and maximize total network information content for a given number of stations, by placing those stations in their optimal locations. Multiobjective optimization provides a robust and defensible means of determining an optimal hydrometric network when considering two or more design objectives. Dual entropy and multiobjective optimization (DEMO) is well established tool that utilizes entropy theory to define optimization objectives and a multiobjective optimization tool to facilitate the optimization process [17]. In this study, the non-dominated sorted genetic algorithm II (NSGAII) [28] was employed. The NSGAII utilizes a fast, non-dominated sorting approach by reducing high computational complexity and elitism, which can speed up the performance of genetic algorithm significantly while help preventing the loss of good solutions once they are found. The NSGAII model parameters in this study are shown in Table 2. The number of the decision variables is equal to the number of the potential stations for each sub-basin, respectively (see Table 1).

The two core design objectives for DEMO are to maximize the total information content of the proposed optimal networks (defined in entropy theory as joint entropy) and minimize the amount of redundant information present (defined in entropy theory as total correlation). This is represented mathematically as:

(6) $$\max\left[H\left(S_{N,M}\right)=H\left(E_{1},E_{2},\ldots ,\;E_{N},\;X_{1},X_{2},\;\ldots ,\;X_{M}\right)\right]\;\min\left[C\left(S_{N,M}\right)=C\left(E_{1},E_{2},\ldots ,\;E_{N},\;X_{1},X_{2},\;\ldots ,\;X_{M}\right)\right]\;subject\;to:N\;and\;M\;are\;integers,\;M\;\in \left\{1,2,\;\ldots ,\;M_{max}\right\}$$

where $S_{N,M}$ is a hydrometric network of *N* existing stations (*E*), and *M* additional stations (*X*). For this study, the maximum number of potential stations was determined by the catchment delineation (see Section 2). However, the number of potential stations to add to the existing network is not pre-defined, but instead is decided during the multiobjective optimization process.

As is characteristic of the multiobjective optimization problems, the output from DEMO is a series of non-dominated optimal solutions, which together form what is referred to as a Pareto-front of solutions. Any optimal solution that lies along the Pareto-front is not dominated by other solutions, meaning that an improvement cannot be made to one of the optimization objective function values without in turn worsening the other objective function value. For the purposes of this study, each point on the Pareto-front represents an optimal hydrometric network.

## 4. Methodology 

**Figure 3:**
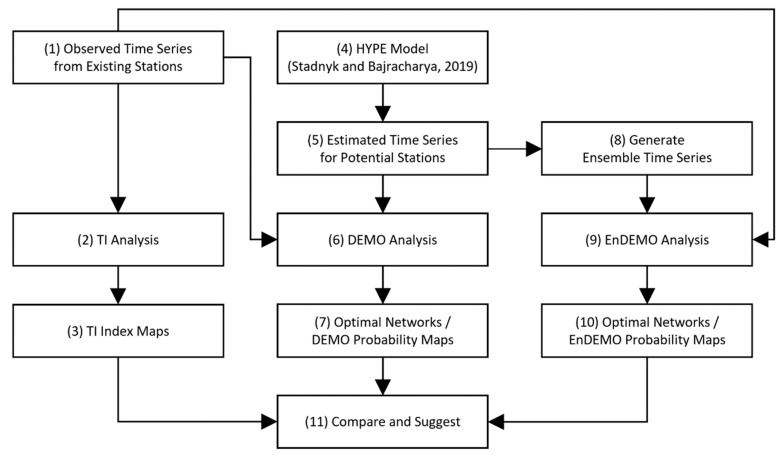
Process diagram of the method.

The process diagram presented in Figure 3 provides a conceptual overview of the methods used in this study. Each major step is indicated in a box of the diagram, and detailed descriptions of the methods follow thereafter.

(1) Obtain daily observed streamflow time series from the existing stations. In this study, the HYDAT database of Environment and Climate Change Canada (ECCC) and the National Water Information System (NWIS) database of the United States Geological Survey (USGS) were used [23].
(2) Estimate the transinformation (TI) index for each existing station to evaluate the current network.
(3) Draw the TI index maps by spatially interpolating the values from (2).
(4) Select a regionalization method or a hydrologic model to generate estimated (or synthetic) time series data at potential station locations. In this study, simulated time series from HYPE model by Stadnyk and Bajracharya (2019) were used.
(5) Obtain the daily estimated runoff time series data for potential stations from (4).
(6) Run the dual entropy and multiobjective optimization (DEMO) tool.
(7) Determine the optimal networks and analyze the networks by creating maps of the station selection frequency.
(8) Generate ensemble runoff time series to account for the model uncertainty in runoff estimation.
(9) Run ensemble-based DEMO (EnDEMO) with the ensemble time series. Ten ensemble members were applied in this study.
(10) Analyze the optimal networks from the EnDEMO application and draw maps of the station selection frequency.
(11) Compare the DEMO with the EnDEMO results and make recommendations.

### 4.1. Ensemble-Based DEMO (EnDEMO)

Simulated time series from a regionalization method or a rainfall-runoff model are prone to model uncertainty arising from model calibration, parameterization, and imperfections, and idealization of the model itself. To account for such types of uncertainty, ensemble streamflow is generated from the simulated time series, and each ensemble is run independently in the DEMO analysis. This analyzing process is referred to as the EnDEMO.

A ten-year window period (2001–2010), which is the recommended time window for the hydrometric network design problems using daily hydrologic time series [14], was selected to provide an estimated daily streamflow time series for the 2693 catchment outlets (i.e., potential stations). For each estimated daily time series from HYPE model output, 1000 ensemble streamflows were generated using Kirsch–Nowak method [29]. The Kirsch–Nowak synthetic streamflow generation method employs two separate processes to generate ensemble streamflows. First, synthetic monthly flows are produced using the Cholesky decomposition method [30]. This process is summarized as follows:

- Daily estimated flows from each subcatchment are aggregated to monthly streamflow time series. The monthly historical streamflows are log-transformed and then normalized by the sample mean and the standard deviation values, so that the resulting standardized log-transformed monthly streamflow time series follow a standard normal distribution.
- Then synthetic ensembles are randomly populated from the standardized log-transformed monthly flows that satisfy its statistics.
- Additional requirements are set when randomly sampling the synthetic ensemble time series. The method of Cholesky matrix decomposition is used to populate monthly synthetic flows that preserve the month-to-month and year-to-year historical autocorrelation matrix of the raw log-transformed streamflow time series.
- The ensemble standardized log-transformed flows are transformed back to real space, ensemble, monthly streamflows by de-standardizing and log-inversing.

Secondly, disaggregation of monthly flows to daily time series is performed by using Nowak’s disaggregation method [31] as follows:

- The k-nearest neighbor is estimated for each ensemble month from all estimated monthly values collected from the surrounding subcatchments using the real space Euclidean distance.
- The k-nearest neighbors in each month are ranked from the nearest to the furthest sites.
- Using a Kernel estimator, the probability of selecting a neighbor is estimated for each site that has an estimated daily time series and the closest neighbor is selected accordingly.
- The final step is to proportionally disaggregate the ensemble monthly streamflow to daily time series using the selected neighboring site.

Among the pool of 1000 daily ensemble streamflow time series, ensemble time series which are not too extreme are chosen to be used in the EnDEMO. In specific, ensemble boundaries were set as two times the standard deviation of the deterministically estimated streamflow time series from the HYPE model. Ten ensemble members appear within the boundaries for 98 percent of the whole time period; therefore, ten ensemble streamflow time series are selected for the EnDEMO analysis. Then, ten traditional DEMO runs are conducted using each member of the ensemble time series respectively. For the EnDEMO analysis, the same values of the NSGAII parameter in DEMO analysis were also used.

### 4.2. Hydrological Prediction for the Environment (HYPE) Model

The HYPE model is a semi-distributed conceptual model adapted from the original Hydrologiska–Byråns–Vattenbalansavdelning (HBV) model by the Swedish Meteorological and Hydrological Institute (SMHI) [32]. The basin is divided into sub-basins, which are further divided into classes, which are smaller computational units. The HYPE model for this study was provided by Stadnyk and Bajracharya (2019). Briefly, the model was constructed by calibrating and validating for 38 gauging locations for the Water Survey of Canada and two additional derived gauges for the Nelson and Churchill River outlets. Readers who are interested in the details of the model construction may refer the original report by Stadnyk and Bajracharya (2019). The simulated discharge from HYPE model for each catchment outlet was used as the runoff of the corresponding potential station location after a proper conversion dividing by drainage area.

## 5. Results and Discussions

### 5.1. TI Index Map

**Figure 4:**
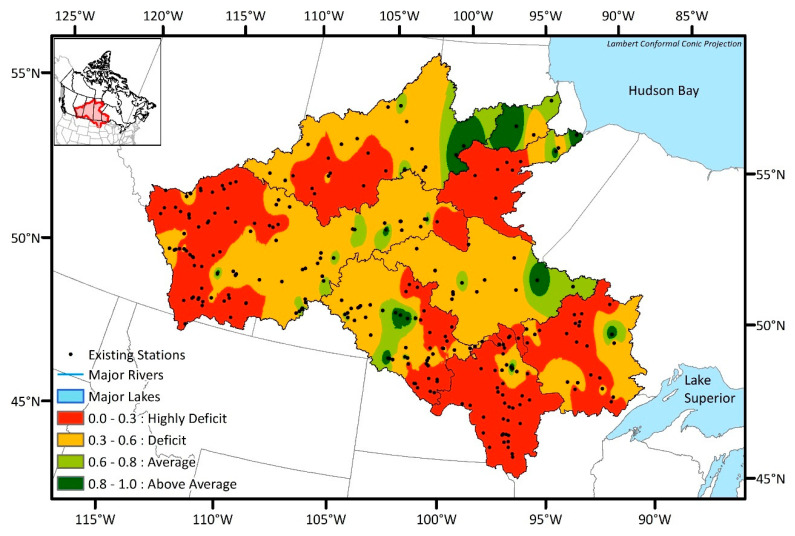
Transinformation (TI) index map.

Transinformation (TI) index was calculated for each existing station using the streamflow observations. The TI index map was then created by spatially interpolating the individual TI index values for each sub-basin. The inverse distance weighting (IDW) method embedded in ArcGIS software was used for the interpolation. Figure 4 shows the TI index map for the NCRB by joining the TI index map for each sub-basin together. The TI indices were categorized into four zones based on the level of information content from the existing hydrometric network. The categories are:

- Highly Deficit: TI index of 0.0 to 0.3;
- Deficit: TI index of 0.3 to 0.6;
- Average: TI index of 0.6 to 0.8;
- Above Average: TI index of 0.8 to 1.0.

Deficit and Highly Deficit zones indicate that stations within these areas have relatively lower TI indexes and share little information with nearby stations. In other words, these stations are more unique and independent with respect to their information content. On the other hand, Average and Above Average zones have stations with relatively higher TI index values and suggest a low priority area because the stations are mutually dependent by having some duplicate information content. Most of the NCRB sub-basins were characterized as Deficit and Highly Deficit TI index categories, which indicate high priority zones due to the greater information deficiency. The majority of the Lower Churchill and downstream areas of Lake Winnipeg, Nelson, and Upper Churchill exhibit an Average or Above Average TI index category and are classified as low priority zones because these regions host stations with relatively higher TI index values. Vast areas of the Red, Winnipeg, and Nelson sub-basins are dominated by Highly Deficit regions, whereas Assiniboine, Lake Winnipeg, Saskatchewan, and Upper Churchill sub-basins have mostly Deficit regions. Overall, these results indicate that seven out of eight sub-basins of the NCRB predominantly have severe information deficits and should be considered high priority areas for future monitoring efforts. The presence of many closely clustered existing stations within the Highly Deficit regions of Saskatchewan and Assiniboine sub-basins shows that while the station density in this region is relatively high, the spatial distribution of the existing stations in this area is sub-optimal. Meaning that despite having high spatial density, the unique information that we can obtain from these stations is very minimal compared to other stations within each sub-basin. In general, the greatest information deficit lies relatively upstream and in the head-water areas of each NCRB sub-basin. Given that the TI analysis is for evaluating the existing hydrometric network, it is anticipated that the DEMO analysis will highlight the probability of selecting an optimum number and location of new hydrometric networks mainly in the Highly Deficit to Deficit zones.

### 5.2. DEMO Results

**Figure 5:**
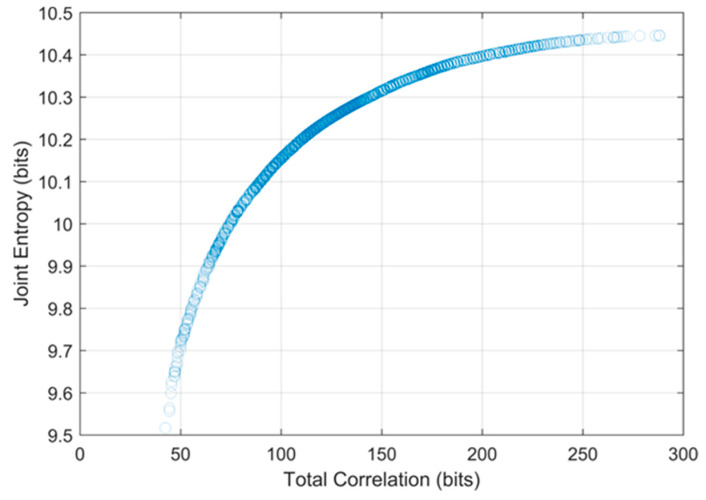
Pareto-front plot from the dual entropy and multiobjective optimization (DEMO) result of the Nelson sub-basin.

To determine the optimal hydrometric networks, the traditional DEMO approach was applied first. Because the DEMO employs a multiobjective optimization tool as a solution searching algorithm, the optimal solutions from DEMO can be many, while allowing trade-offs between objectives, and each optimal solution cannot be dominated by any other optimal solutions. Figure 5 shows the Pareto-front, which indicates objective values of optimal solutions from the DEMO result of the Nelson sub-basin. While the entropy values from a dataset can change due to the quantization method, having too high (i.e., close to the maximum entropy) or too low (i.e., close to zero) entropy values in optimal networks can be less reliable. Considering that the maximum entropy of the given time series (3652 days), which is called as the saturated entropy, is log_2_ 3652 = 11.83 bits, the range of the joint entropy of the optimal solutions, which is from 9.5 bits to 10.5 bits is appropriate. The number of the selected stations in each optimal network in the Nelson sub-basin ranges from 33 to 190 and total correlation varies from 42.45 bits to 288.14 bits. In general, total correlation is lower in the Pareto-front when the number of the selected stations is lower and vice versa. The shapes of the Pareto-front for other sub-basins were similar that of the Nelson sub-basin, so they are not presented here. Instead, Table 3 shows the number of the optimal networks, ranges of the number of the selected stations in the optimal networks, and their objective values for each sub-basin.

**Figure 6:**
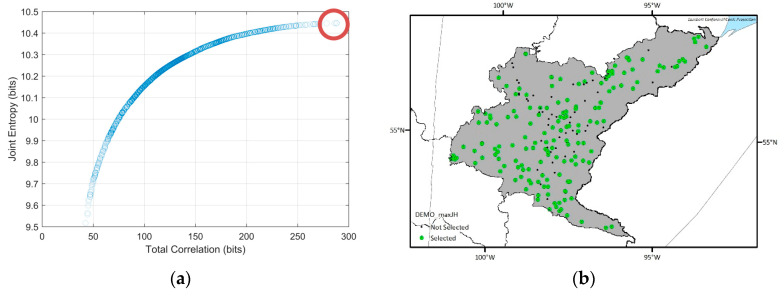

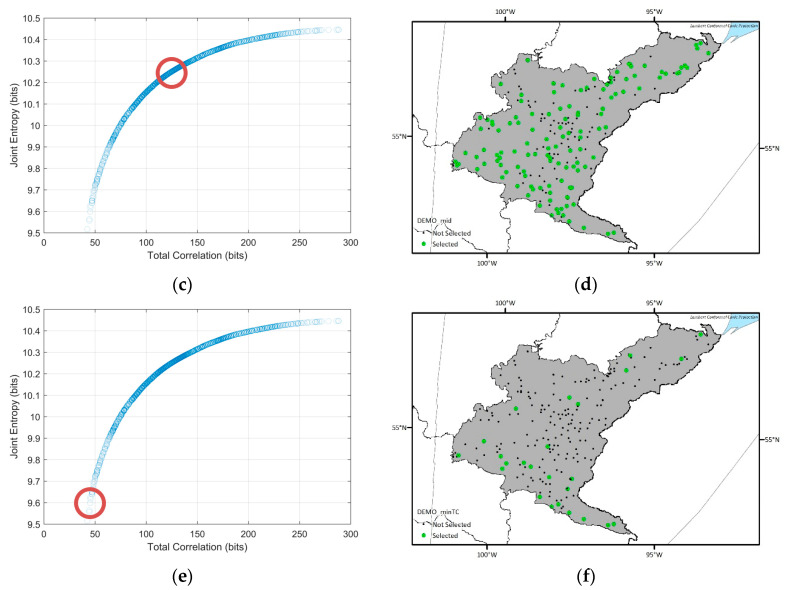
Spatial distributions of the selected optimal networks and their locations (red circles) in the Pareto-front for the Nelson sub-basin. (**a**,**b**) Optimal network which has the maximum joint entropy among the optimal solutions; (**c**,**d**) optimal network which has the median joint entropy and total correlation values among the optimal solutions; (**e**,**f**) optimal network which has the minimum total correlation among the optimal solutions.

**Figure 7:**
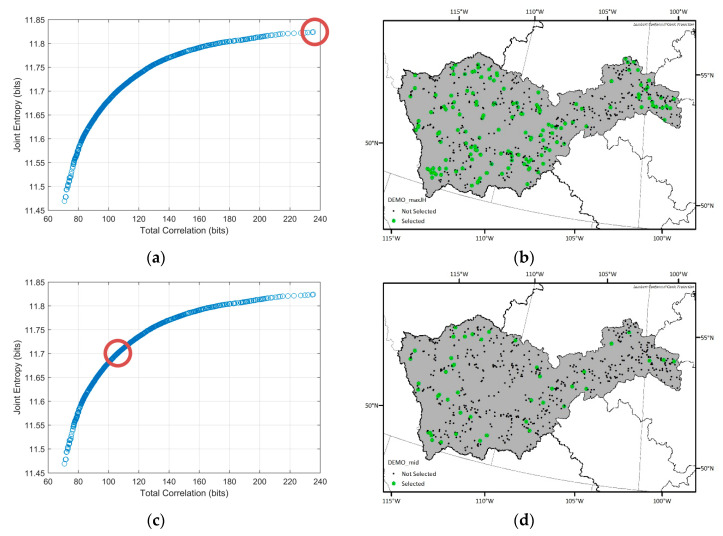

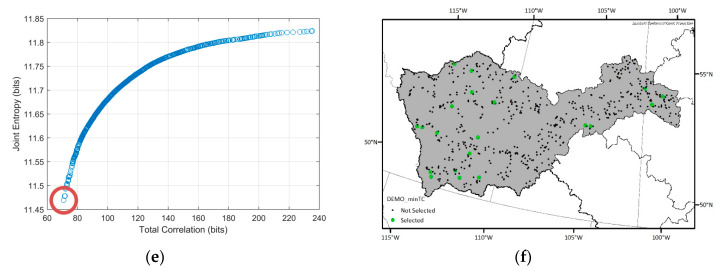
Spatial distributions of the selected optimal networks and their locations (red circles) in the Pareto-front for the Saskatchewan sub-basin. (**a**,**b**) Optimal network which has the maximum joint entropy among the optimal solutions; (**c**,**d**) optimal network which has the median joint entropy and total correlation values among the optimal solutions; (**e**,**f**) optimal network which has the minimum total correlation among the optimal solutions.

**Figure 8:**
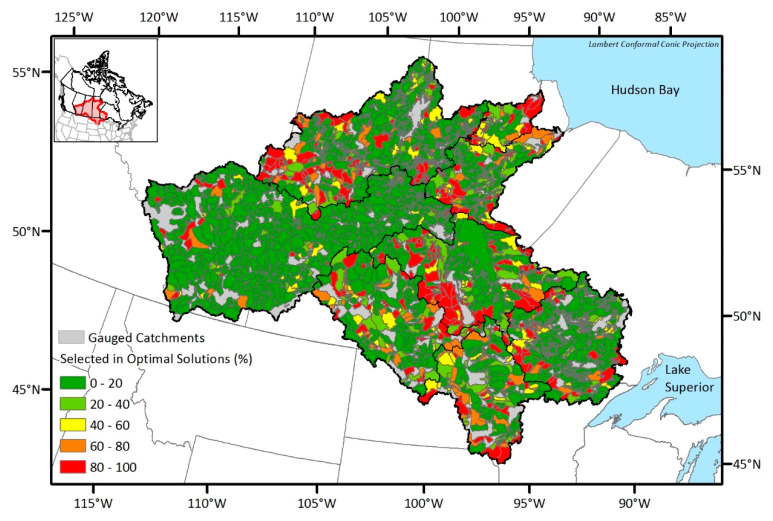
Map of the station selection frequency from the DEMO results.

Considering that there are many optimal networks (i.e., 454 to 1415) from each DEMO run, presenting all the optimal solution is not practical. Rather, spatial distributions of the selected stations in three example solutions of Nelson and Saskatchewan sub-basin which are the solutions having maximum joint entropy, minimum total correlation, and median objective values are shown in Figure 6 and Figure 7, respectively. The number of selected stations in the optimal solutions are high in the maximum joint entropy solutions and low in the minimum total correlation solutions. Since those are all the optimal solutions, it would be the best if the network managers choose an optimal solution and installed new stations at once. However, because installing stations highly depends on the budget, even the minimum numbers of the additional stations in the optimal networks vary from six to 70 respectively, and the network manager should select a network among the Pareto-front optimal solutions considering scientific and socioeconomic needs, it is sometimes not achievable to establish an optimal network. Therefore, we suggest a map of the station selection frequency that shows which potential locations more frequently appear in the optimal solutions to support the decision making process in the network design problems. In Figure 8, the drainage catchment of each potential location was shaded by its selection frequency in the optimal networks. For example, if a station has been selected 85 times within the optimal networks, and the number of total optimal networks is 100, the probability is then 85 percent, which is shown in red on the map of the station selection frequency. Therefore, the reddish areas (red or orange) are recommended to be considered as new monitoring locations preferentially. The map of the station selection frequency shown in Figure 8 is a combined map from the respective optimal solutions for each sub-basin.

### 5.3. Uncertainty Considerations using EnDEMO

The EnDEMO concept has been proposed here to account for the uncertainty in the streamflow estimation that is one of the essential processes in many network design problems. As there are ten ensemble streamflow sets which are within the boundary of two times the standard deviation, the EnDEMO run for each sub-basin in this study consists of ten DEMO runs for the respective ensemble members. The map of the station selection frequency from the EnDEMO application were drawn by calculating the individual selection probabilities in the optimal solutions after the ten ensemble sets of the optimal networks were aggregated. Therefore, the map of the station selection frequency from the EnDEMO results can be regarded as a mean frequency from the ten ensemble DEMO runs.

**Figure 9:**
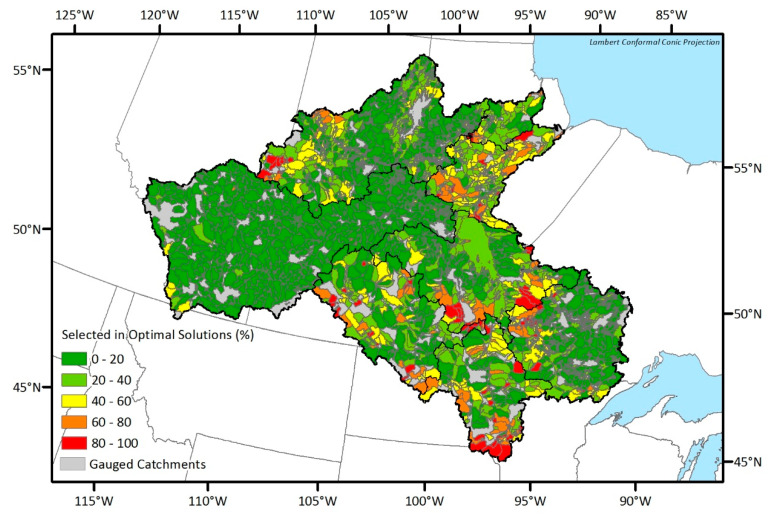
Map of the station selection frequency from ensemble-based dual entropy and multiobjective optimization (EnDEMO) results.

If we compare the map of the station-selection frequency from the EnDEMO results (see Figure 9) with that from the traditional DEMO results (see Figure 8), it is obvious that the number of red and orange areas corresponding to frequently selected locations in the optimal networks are reduced. For example, catchments in the west of the Upper Churchill sub-basin, and near the border between Assiniboine and Lake Winnipeg sub-basins, were mostly red from the DEMO results, while only few of them remained red after considering the input uncertainty. There are catchments which were already red or orange in DEMO results and remained red or orange in the EnDEMO results. On the other hand, there are also some catchments which were green both in the DEMO and the EnDEMO results. Since the changes in color between the two results arose from the uncertainty considerations, the EnDEMO-based results can be regarded as more reliable and robust. Catchments in red or orange from both DEMO and EnDEMO results should be considered the preferential locations for new stations. Conversely, catchments in green from both results should be viewed as the least interesting (or secondary) locations for adding new stations. 

It should be noted that gathering highly selected stations from Figure 8 or Figure 9, the resulting network cannot be guaranteed to be an optimal one, as some stations may belong to different optimal networks. Therefore, if a decision maker wants to select locations, she/he can get information from Figure 8 or Figure 9 on which locations he/she should focus on. 

## 6. Conclusions

This study consists of (1) evaluating the existing networks by TI analysis, (2) determining the optimal networks using DEMO applications, and (3) improving the network reliability by implementing newly proposed EnDEMO method which accounts for uncertainty in the synthetic time series data at potential station locations. A TI index map created by TI analysis highlighted the critical regions where the information content in the existing stations are more unique and independent. Even though there are 267 existing stations in the NCRB, most of the basin was classified as Highly Deficit to Deficit, and this result matched well to the fact that the study area requires more stations based on the WMO’s minimum station density recommendation.

While the TI analysis provided a preliminary overview of the information contents from the existing hydrometric network, the actual optimal network design by determining the optimal locations of the new stations was conducted by applying and comparing DEMO and EnDEMO. Due to the number of potential station locations and the implementation of multiobjective optimization in DEMO, having a number of optimal solutions which ranges from 454 to 1415 for each sub-basin in NCRB was unavoidable. Since investigating all the optimal networks is not practical, maps of the station selection frequency were created to draw a general conclusion from the optimal solutions. The probability of each potential location was calculated by the selection frequency among the optimal solutions, so that the frequently selected stations could be regarded as the locations where the monitoring efforts are primarily needed.

In the DEMO applications, the estimated runoff for the potential stations was obtained from the outputs of a hydrological model (HYPE). The simulated time series inherently have some uncertainty due to model calibration, parameterization, and regionalization processes. Therefore, EnDEMO approach was proposed such that the input runoff time series were perturbed within a two standard deviation range from the deterministically simulated. After comparing the map of the station selection frequency of EnDEMO with that of DEMO, some potential stations showed similar probabilities, while others did not. Therefore, in terms of reliability, it is preferable to consider potential stations where the selection probability in the optimal networks was high in both DEMO and EnDEMO applications. On the other hand, if a potential station was rarely selected in both applications, it would have less priority. In summary, DEMO itself has been successfully implemented in many network design problems; however, the EnDEMO approach was able to provide more robust outcome by (1) including ensemble generation to account for the uncertainty in simulated time series datasets, and (2) identifying the more reliable locations for potential stations.

## Figures and Tables

**Table 1:** List of sub-basins in NCRB and their numbers of existing and potential stations.

Sub-Basins	Numbers of Existing Stations	Numbers of Potential Stations
Assiniboine	54	271
Red	41	218
Winnipeg	25	377
Lake Winnipeg	14	294
Saskatchewan	98	695
Nelson	9	219
Upper Churchill	23	556
Lower Churchill	3	61

**Table 2:** Non-dominated sorted genetic algorithm II (NSGA-II) Parameters.

Model Parameters	Parameter Value
Population Size	3000
Maximum Generations	6000
Number of Decision Variables	Number of Potential Stations (N)
Crossover Operator	Single Point Crossover
Crossover Probability	1.0
Mutation Operator	Bit String Mutation
Mutation Probability	2/N
Variable Type	Binary

**Table 3:** DEMO results.

Sub-Basins	Numbers of Optimal Networks	Numbers of Selected Station Range	Joint Entropy Range (bits)	Total Correlation Range (bits)
Assiniboine	1343	29–219	10.57–11.39	37.76–227.91
Red	1191	19–179	9.22–10.78	19.03–158.54
Winnipeg	1134	34–228	10.95–11.47	57.31–333.89
Lake Winnipeg	1415	60–262	10.87–11.63	65.66–344.52
Saskatchewan	685	22–162	11.47–11.82	70.80–235.38
Nelson	1205	33–190	9.52–10.45	42.45–288.14
Upper Churchill	1036	70–319	10.99–11.54	82.35–456.01
Lower Churchill	454	6–57	5.24–7.25	5.13–76.48
